# Quantifying Clinical HPV4 Dose Inefficiencies in a Safety Net Population

**DOI:** 10.1371/journal.pone.0077961

**Published:** 2013-11-06

**Authors:** Diane M. Harper, Inge Verdenius, Felicia Ratnaraj, Anne M. Arey, Beth Rosemergey, Gerard J. Malnar, Jeffrey Wall

**Affiliations:** 1 Center of Excellence, Women’s Health, University of Missouri–Kansas City School of Medicine, Kansas City, Missouri, United States of America; 2 Department of Biomedical and Health Informatics, University of Missouri–Kansas City School of Medicine, Kansas City, Missouri, United States of America; 3 Department of Obstetrics and Gynecology, University of Missouri–Kansas City School of Medicine, Kansas City, Missouri, United States of America; 4 Department of Community and Family Medicine, University of Missouri–Kansas City School of Medicine, Kansas City, Missouri, United States of America; 5 Department of Obstetrics and Gynecology, Radboud University, Nijmegen, The Netherlands; IPO, Inst Port Oncology, Portugal

## Abstract

**Purpose:**

HPV4 is the most expensive vaccine requiring three appropriately timed doses to provide maximal efficacy against two oncogenic HPV types. The primary purpose of this study is to quantify the use of HPV4 vaccine in a safety net health care system in terms of its inefficiencies.

**Methods:**

A retrospective study of HPV4 dosing from 2006–2009, among females 10–26 years old who sought care in a safety net health care system was conducted to determine dose usage patterns among those at highest risk for cervical cancer. Dose descriptors abstracted from the electronic medical record (EMR) included timing and number in series as well as characteristics of the person to whom and visit at which the dose was given. Dose inefficiencies were separated into “less than three doses” and “mistimed doses” for analysis.

**Results:**

The majority (66%) of HPV4 doses administered were insufficient to induce the maximal immune response necessary for HPV infection prevention. Among on-time doses, 58% were singleton or doublet doses. Mistimed doses accounted for 19% of all doses administered with late intervals being more common than early intervals among those receiving more than one dose (9% vs. 4%, p<0.001). Third doses were mistimed twice as often as second doses (10% vs. 5%, p<0.001). Black women were more likely to have a mistimed second dose and Hispanic women more likely to have a mistimed third dose compared to white women (OR = 1.70 (95% CI: 1.11, 2.61 and 2.44 (1.19, 5.00), respectively). The HPV4-only visit type at which HPV4 was initiated was the most significant predictor of on-time doublet completion.

**Conclusions:**

In a safety net health care system the large inefficiencies associated with HPV4 vaccination must be addressed in order to maximize our patient’s cervical cancer prevention.

## Introduction

Currently, at $135.45 per dose retail ($98.60 per dose via the Centers for Disease Control and Prevention (CDC)) [1], HPV4 is the most expensive prophylactic vaccine marketed. Administrative, storage and programmatic tracking costs add to the expense of HPV4 vaccination [2]. The CDC purchased $51 million of HPV4 for the Strategic National Stockpile [3] and continues to purchase 10 million doses of HPV4 yearly [4], yet there is little evidence of appropriate use of this resource among those at highest risk for cervical cancer.

Because the completion rates of the on-time three-dose HPV4 series after initiation are low, ranging from 12–37% within the US [5]–[25], one focus of research has been to evaluate a two dose regimen [26]–[28]. While there are no efficacy data to support less than three doses of HPV4, and there are no registered studies of memory B-cell responses for less than three doses, the antibody data show that two doses of HPV4 induce inferior antibody titers for HPV 6 and HPV 18 compared to three doses within three years of vaccination [26]–[28]. This may lead to reduced efficacy, reduced duration of efficacy, and hinder the cost effectiveness of the prophylactic HPV vaccine program for cervical cancer prevention [29].

Historically, other three dose vaccines have a moderately low 40–45% on-time completion rate for adolescents and young adults, with only 1–2% receiving mistimed doses [30]. However, the vaccine costs were one tenth the cost of HPV4 [1], mitigating the economic misuse of resources. The purpose of this study was to quantify the use of HPV4 vaccine in a safety net health care system in terms of its inefficiencies. The inefficiencies are classified as “less than three doses” administered and as “mistimed doses”.

## Methods

This research was approved by the Truman Medical Center (TMC) Privacy Board and by the University of Missouri Kansas City (UMKC) Adult Health Sciences Institutional Review Board as an exempt study not requiring individual consent (#11–16e).

TMC provides care to vulnerable uninsured, underinsured and low income patients at high risk for adverse outcomes such as cervical cancer [31], [32]. The HPV4 vaccination program was started in July 2006 and remained unopposed until October 2009 when HPV2 was added to the program. Descriptors about the HPV4 doses were abstracted from the electronic medical record (EMR) during this unopposed time frame. Only female recipients 10–26 years old were targeted for vaccination. Prior research identified age, race, and parity as positive predictors of on-time three dose completion [6], and hence these person descriptors were included in this study.

Dose descriptors include the number order in which each dose was received and date of its administration. Prior research also identified clinic and systems-level factors surrounding the health care visit as important to timely series completion. For this study, the type of visit at which HPV4 was administered was coded as an HPV4-only visit where a standing order for HPV4 was available for females meeting a specific set of criteria; or a health care visit for acute needs, follow up from an acute visit, a preventive visit, a visit either immediately postpartum or within the six week interval thereafter or ‘other’ visit.

Mistimed doses were classified as in previous publications as a dose given too early or too late from the prior dose [6]. The early intervals were defined as less than 4 weeks between dose 1 and dose 2; less than 12 weeks between dose 2 and dose 3; or less than 24 weeks between dose 1 and dose 3. Late intervals were defined as more than 26 weeks between dose 1 and dose 2; or more than 52 weeks between dose 1 and dose 3 [6], [26]–[28], [33], [34]. Up to two mistimed doses could be recorded for each woman initiating vaccination. Dose mistiming was also identified as a fourth dose when a fourth dose was given regardless of whether three of the four doses were appropriately timed.

### Statistics

Analyses were performed on a per dose basis with time linkage to the woman receiving the dose. Descriptive statistics included means testing by one-way analysis of variance and t-testing using a two sided alpha of 0.05 and Bonferroni corrections for multiple comparisons. Chi-square testing was used for comparisons of ratios. The Cochrane-Armitage test for trend was used when more than two proportions were compared. As HPV2 has already been proven to be efficacious in less than three doses [35], we anticipate that a budgetary shift to two dose HPV vaccination will be adopted by those with limited resources. Hence, we used binary logistic regression analyses to evaluate the predictors of doublet dosing. Timing of dosing will continue to be important, whether for a doublet or triplet dose schedule; and hence binary logistic regression was used to highlight the differences in predictors of mistimed doublet vs. mistimed triplet doses. All data analyses were performed using Statistica v9.1 [36].

## Results

Our work shows that 66% (1967/2993) of HPV4 doses were administered in a manner that cannot provide the long term efficacy to reduce abnormal Pap screenings, colposcopies and treatment procedures as was shown for three on-time doses as approved by the FDA [37]. Of the 2993 doses reviewed in this study, 651 (22%) doses were given as singleton doses, 818 (27%) as doublet doses, 1464 (49%) as triplet doses and 60 (2%) as quadruplet doses. Both insufficient numbers of doses as well as mistimed doses contributed to the inefficiencies.

### Number of Doses

In order to separate the effect of “less than three doses” from “inappropriate timing of doses”, we only considered doublets and triplets given at appropriate time intervals for our ‘number of doses’ analysis. This results in a distribution of 651 (27%) singletons, 745 (31%) on-time doublets and 1026 (42%) on-time triplets among both triplet and quadruplet doses (**Table 1**).

**Table 1:** Descriptors by number of appropriately timed doses.

		Singleton	Doublet	Triplet
		N = 651	N = 745	N = 1026
**Characteristics of Person receiving HPV4 dose**				
**Age, yrs**		**mean (SD)**	**mean (SD)**	**mean (SD)**
All		**21.3 (3.0)**	**20.8 (3.5)**	**20.4 (3.8)**
10–17 year		**15.8 (1.6)**	**15.2 (1.9)**	**14.8 (2.3)**
18–26 years		22.0 (2.4)	21.9 (2.5)	22.0 (2.4)
**Race** †		**n (%)**	**n (%)**	**n (%)**
White		223 (35.2)	243 (32.6)	423 (41.2)
Black		359 (56.6)	381 (51.1)	525 (51.2)
Hispanic		**52 (8.2)**	**83 (11.1)**	**27 (2.6)**
**Gravidity**		n = 605	n = 684	n = 980
n = 0		494 (81.7)	537 (78.5)	**564 (55.0)**
**Parity**		n = 605	n = 684	n = 980
n = 0		467 (77.2)	519 (75.9)	**516 (50.3)**
**Characteristic of the Visit at which HPV4 was initiated**				
HPV4 alone		**324 (49.8)**	**464 (62.3)**	**713 (69.5)**
Health Care visit		327 (50.2)	281 (37.7)	313 (30.5)
	Acute Illness	57 (8.8)	51 (6.8)	73 (7.1)
	Follow up from acute illness	**8 (1.2)**	**15 (2.0)**	**42 (4.1)**
	Preventive visit	44 (6.8)	50 (6.7)	89 (8.7)
	Postpartum	**192 (29.5)**	**76 (10.2)**	**52 (5.0)**
	Other*	26 (4.0)	31 (4.2)	57 (5.5)
**Year Administered**				
	2007	72 (11.1)	101 (12.3)	**222 (21.6)**
	2008	318 (48.8)	409 (54.9)	527 (51.4)
	2009	**260 (39.9)**	**232 (31.1)**	**252 (24.6)**
	2010	1 (0.2)	3 (0.4)	**24 (2.3)**

Singleton means only one dose was administered; doublet means that two doses were appropriately timed; triplet means that three doses were appropriately timed.

Bold font signifies significant differences among singleton, doublet and/or triplet doses at p<0.001.

* Doses administered at visits at which counseling for smoking cessation, depression, or contraceptive use; and procedures including IUD placement occurred.

† Doses administered to Other races than White, Black and Hispanic make up 4% of singleton and doublet doses and were not included in this table.

Increasing numbers of doses occurred at significantly younger mean ages among the 10–17 year olds: mean age associated with a singleton dose was at 15.8 yrs (SD 1.6) decreasing to 15.2 yrs (1.9) for doublet doses and to 14.8 years (2.3) for triplet doses (p<0.006). Only Hispanic race was significantly associated with singleton (8%) and doublet doses (11%) compared to triplet doses (3%) (p<0.001); white and black races were equally distributed among all three dose numbers. Nulligravid and nulliparous recipients occurred significantly more often with singleton (82% and 77%, respectively) and doublet (79% and 76%) doses than with triplet dosing (55% and 50%, p<0.001).

The type of visit at which the HPV4 dose was initiated was significantly associated with doublet and triplet completion. Specifically, initiating the HPV4 series at a HPV4-only visit or a follow up from an acute illness visit was significantly associated with increasing numbers of doses. The percentages of visits at which a singleton, doublet and triplet dose was initiated at a HPV4-only visit were 50%, 62% and 70% (p<0.001), respectively; and at a follow up from an acute illness visit were 1%, 2% and 4% (p<0.001), respectively. The opposite significant trend with less doses completed occurred if HPV4 was initiated at a postpartum visit: 30%, 10%, 5% (p<0.001) for singletons, doublets and triplets, respectively. Initiating HPV4 vaccination at an acute illness or a preventive visit showed similarly equal associations with singleton, doublet and triplet completion.

Six months after HPV4 received federal regulatory approval, the first HPV4 dose in our safety net health care system was administered. Of the doses administered in 2007, significantly more triplets occurred than singleton and doublets (22% vs. 11% vs. 12%, p<0.001); and, likewise for 2010 (2% vs. 0.2% vs. 0.4%, p<0.001). The year 2008 showed an even distribution of singleton, doublet and triplet dose administration: 49%, 55% and 51%, respectively. The year 2009 showed significantly more singleton and doublet doses than triplet doses (40% vs. 31% vs. 25%, p<0.001).

It is highly unlikely that a single dose of HPV4 will ever achieve the protective efficacy that three doses has been proven to accomplish. However, HPV2 does have efficacy data for two doses [35], and recent HPV4 work intimates possible sustained anti-HPV 16 titers for 3 years after only two doses [26]. Hence, understanding predictors of two dose compliance becomes important. **Table 2** shows that multivariate regression predicts an 8% increase in on-time doublet dose compliance with older females compared to younger girls (aOR = 1.08 95% CI: 1.01, 1.16). Most striking was the importance of the HPV4 initiation visit. When HPV4 was initiated at a preventive visit or at a postpartum visit, an on-time second dose was less likely to occur than if the initiation visit had been a HPV4 only visit (aOR = 0.18 (0.09, 0.36) and. aOR = 0.37, (0.20, 0.69), respectively).

**Table 2:** Predictors of Appropriately Timed Doublet Dosing.

	Crude OR (95% CI)	Adjusted† OR (95% CI)
**Characteristics of Person receiving HPV4**		
**Age (10–26 years)**	**1.07 (1.00, 1.14)**	**1.08 (1.01, 1.16)**
**Race/Ethnicity**		
White	referent	
Black	0.69 (0.39, 1.22)	
Hispanic	0.93 (0.38, 2.28)	
**Gravidity**		
n = 0	referent	
n≥1	0.82 (0.47, 1.44)	
**Parity**		
n = 0	referent	
n≥1	0.83 (0.48, 1.43)	
**Visit Type at first dose**		
HPV4 alone	referent	referent
Acute Illness	0.48 (0.19, 1.21)	0.44 (0.17, 1.12)
Follow up from acute illness	0.42 (0.09, 1.94)	0.39 (0.08, 1.79)
Preventive Visit	**0.19 (0.09, 0.38)**	**0.18 (0.09, 0.36)**
Postpartum	**0.38 (0.20, 0.69)**	**0.37 (0.20, 0.69)**
Other*	0.43 (0.14, 1.32)	0.42 (0.14, 1.29)

N = 785 second on-time doses administered.

† Adjusted for significant univariate characteristics: visit type and age.

* Counseling for smoking cessation, depression, or contraceptive use; and Procedures including IUD placement.

Bold font values indicate significance.

### Dose Timing

On-time dosing was significantly higher among doublet doses (91%) than among triplet (69%) or quadruplet (35%) doses (p<0.001) shown in **Table 3**. 13% (299/2342) of doses are mistimed from at least one other dose in either an early or late manner. Among triplets 6% (83/1464) of third doses were administered more than 365 days after the first dose.

**Table 3:** Dosing intervals by dose number and timing status.

	Doublet	Triplet	Quadruplet
	N = 818	N = 1464	N = 60
On-Time†	745 (91%)	1005 (69%)	21 (35%)
Early Dosing			
Dose 2 from Dose 1	0 (0%)	2 (<1%)	2 (3%)
Dose 3 from Dose 2		27 (2%)	2 (3%)
Dose 3 from Dose 1‡		56 (4%)	4 (7%)
Late Dosing			
Dose 2 from Dose 1	73 (9%)	46 (3%)	1 (2%)
Dose 3 from Dose 1		83 (6%)	3 (5%)
Two Dosing Intervals Inappropriately Timed		20 (1%)	2 (3%)
Extra doses after a mistimed three dose series			32 (53%)
Extra doses after an appropriately timed three dose series*			7 (12%)

†Significant difference among doublet, triplet and quadruplet on-time doses, p<0.001.

‡Early interval of dose 3 from dose 1 occurred significantly more often than other early dosing intervals, p<0.002.

* Six of the seven extra doses were prior to the appropriately timed triplet, and one of the seven was after the appropriately timed triplet.

Among mistimed doses, significantly more were too late compared to too early (9% (206/2342) vs. 4% (93/2342), p<0.001); and 1.7% (39/2342) were fourth doses associated with either a mistimed triplet or were superfluous to the completed on-time triplet. The mean number of days too late for dose 2 after dose 1 was 388 (SD 194) days; and for dose 3 after dose 1 was 577 (SD 202) days. The proportion of late intervals between dose 3 and dose 1, 5.6% (86/1524), was no different than the proportion of late intervals between dose 2 and dose 1, 5.1% (120/2342).

The mean number of days too early between dose 1 and dose 2 was 8.0 (SD 5.3) days, between dose 1 and dose 3 was 13.2 (SD 13.4) days and between dose 2 and dose 3 was 17.5 (SD 19.1) days. Dose 3 occurred significantly too early from dose 1 more often than dose 2 was too early from dose 1, or dose 3 from dose 2 (4% (60/1524) vs. 0.2% (4/2342) vs. 1.9% (29/1524), p<0.002, respectively). Similarly dose 3 was too early from dose 2 significantly more often than dose 2 was too early from dose 1 (1.9% (29/1524) vs. 0.2% (4/2342), p<0.001).

Overall, third doses are mistimed significantly more often than second doses (11% (175/1524) vs. 5% (124/2342), p<0.001). In addition, predictors of mistimed second and third doses are different (**Table 4**). Mistimed second doses occur significantly more frequently in younger vaccinees than older (OR = 0.94 95% CI: 0.90, 0.99), and in blacks more frequently than whites (1.70 (1.11, 2.61); whereas, mistimed third doses occur significantly more frequently among Hispanics than whites (2.44 (1.19, 5.00)). The adjusted model provides no further insight, except that mistimed third doses are more likely to happen among both black and Hispanic women than among white women.

**Table 4:** Predictors of Mistimed Doses.

	Mistimed 2^nd^ Dose		Mistimed 3^rd^ Dose	
	OR (95% CI)	aOR‡ (95% CI)	OR (95% CI)	aOR‡ (95% CI)
**Age**	**0.94 (0.90, 0.99)**	**0.94 (0.89–0.99)**	0.97 (0.93, 1.01)	0.97 (0.93–1.02)
**Race**				
White	Referent	Referent	Referent	Referent
Black	**1.70 (1.11, 2.61)**	**2.14 (1.38–3.34)**	1.31 (0.90, 1.90)	**1.52 (1.03–2.24)**
Hispanic	1.66 (0.77, 3.56)	1.94 (0.89–4.22)	**2.44 (1.19, 5.00)**	**2.80 (1.35–5.79)**
**Gravidity**				
n = 0	Referent			
n≥1	0.86 (0.58, 1.27)	§	0.97 (0.68, 1.37)	§
**Parity**				
n = 0	Referent	§		§
n≥1	0.79 (0.53, 1.16)		0.91 (0.64, 1.28)	
**Visit Type at first dose**				
HPV4-only	Referent	Referent	Referent	Referent
Other Health Care Visit Types	**3.32 (2.28, 4.83)**	**3.53 (2.39–5.23)**	**1.87 (1.33, 2.62)**	**1.97 (1.39–2.80)**
**Visit Type at first dose**				
HPV4-only	Referent	§		§
Acute Illness	1.85 (0.89, 3.86)		1.18 (0.59, 2.36)	
Follow up from acute illness	**3.81 (1.73, 8.39)**		1.08 (0.42, 2.79)	
Preventive Visit	**3.97 (2.39, 6.60)**		**2.96 (1.90, 4.62)**	
Postpartum	**3.90 (2.37, 6.40)**		1.52 (0.78, 2.97)	
Other*	**2.71 (1.29, 5.69)**		1.83 (0.93, 3.60)	

N = 124 mistimed second doses among all doses delivered; N = 153 mistimed third doses among all doses delivered.

* Counseling for smoking cessation, depression, or contraceptive use; and Procedures including IUD placement.

‡adjusted for significant variables in univariate model.

§Not included in multivariate model due to lack of significance or co-linearity.

Bold font indicates a significant predictor.

Both mistimed second and third doses occur more frequently when HPV4 initiation occurred at visits at which other health care was provided compared to HPV4-only visits (OR = 3.32 (95% CI: 2.28, 4.83) and 1.87 (1.33, 2.62), respectively). In our population, initiating HPV4 vaccination at a preventive visit is about three times more likely to result in a mistimed second or third dose (OR = 3.97 (95% CI: 2.39, 6.60) and 2.96 (1.90, 4.62), respectively). A mistimed second dose is equally and significantly influenced by initiating HPV4 vaccination at a follow up from an acute visit, a postpartum visit, or any other health care visit as at a HPV4-only visit.

## Discussion

Although there have been millions of doses of HPV4 sold every year [38], [39], our study and others [6], [8]–[25], show that 66% of the doses are administered in an inefficient manner, making the HPV4 vaccination program unlikely to realize a measurable benefit especially in populations most at-risk, such as our safety net system. Of the inefficiencies, “less than three doses” was more common than inappropriate timing of doses.

Health economic studies show that limited duration of vaccine efficacy and low female population coverage result in HPV vaccination programs not being cost-effective on a population level [29], [40]. Insufficient numbers of and mistimed HPV4 doses have had little rigorous attention [41] but substantially and negatively impact the cost effectiveness of the vaccination program [29], [40], [42]–[44]. Receiving fewer than three doses in other three dose series is recognized by the Office of Inspector General as a common and costly issue for the Vaccine for Children (VFC) program [45]. The vast majority of our doses were administered as singleton or doublet doses. Maximizing health care resources in the near term may indicate a shift to the use of HPV2, the bivalent HPV vaccine. It has efficacy proven for both one or two doses, despite FDA labeling for three doses, lasts at least 4 years for less than three doses, and may protect against seven oncogenic HPV infections at varying efficacies [35], [46], [47] Our study showed that two doses were highly likely to be completed on-time and more likely if provided at a vaccine-only visit than at a preventive visit.

Mistimed dosing, while occurring less often than insufficient doses, contributed a substantial portion of inefficient dosing. Studies of HPV4 and other vaccines to date have shown that compliance with dosing intervals is critical for the induction of immune responses and long term memory [26]–[28], [48], [49]. Early and late dosing deviations have been noted for the two dose series of Varicella and Hepatitis A and the three dose series of Hepatitis B vaccinations in similar age ranges [30]. Early dosing intervals occurred more commonly for HPV4 in our study, than for Varicella, Hepatitis A or Hepatitis B vaccines in other studies [30]; but late dosing of the second or third dose in our study occurred similarly for HPV4 as for the reference vaccines [30].

Inefficient use of HPV4 doses in a safety net health care system is distressing from many perspectives [50]. Less than three doses occurred more frequently as time moved forward in our study for reasons potentially attributed to less mass media advertising, less enthusiasm on the part of the medical professionals and increased publicity of possible rare side effects. In addition, HPV4 is the most costly vaccine ever to be commercialized and hence puts an economic burden on the public financing structures as well as insurance companies that underwrite the majority of doses dispensed for this population. The large numbers of girls and women who have received insufficient doses or mistimed HPV4 dosing may not understand that their HPV4 doses are not proven to prevent HPV infection, much less pre-cancerous disease. Moreover, these females who are already vulnerable and at high risk for cancer outcomes may incorrectly assume that they are protected from cervical cancer and do not need to partake in the recommended routine cytology screening program [51]. Lastly, the cost to the physician, immunization providers and safety net health system in terms of interruptions in cold chain maintenance, clinical scheduling logistics, vaccine administration and expiration wastage, and immunization support and programmatic office costs can be nearly as much as the cost of the vaccine itself [52].

In response to these analyses, we have initiated an HPV2 two dose system with the second dose administered after 4 weeks and before 6 months in our health care system, as there is proven high efficacy for two HPV2 doses so administered [35]; and, our doublet analysis indicates that 91% of these doses are received on time.

### Limitations of the Study

In order to determine whether doses were mistimed, hard criteria for cut-off values had to be established. While some may suggest different cut-off intervals, we used those intervals established by the CDC, by what had been used by other authors, and from newer data to support immune efficacy. Certainly as cut-off thresholds change, the proportions of mistimings may change, but the overall sense that early and late violations will lead to diminished efficacy remains.

Our unit of measure of this study was the dose and its interval from those in the recommended three dose series. Our concern is to determine what characteristics cause failure and success in an appropriately timed three dose series in the hopes of HPV4 vaccination being cost effective. Other authors have used the woman as the unit of measure when determining behavioral compliance with three dose adherence. Our study is not a study of behavioral compliance, but rather a quantification of inefficiencies associated with HPV4 vaccination.

The data source for this retrospective study was the EMR. While information may have been omitted from the EMR, the actual data abstraction was double-checked for accuracy. In addition, it is unlikely, but possible, that females usually served in our catchment area completed their HPV4 series at other health care clinics.

## Conclusions

The inefficiencies of HPV4 administration are very high in our safety net health care system. Cervical cancer prevention programs have been very successful with the current cytology and HPV screening tests in place bringing the incidence of cervical cancer to 6.5/100,000 in our American Indian/Alaska Native women, 7.1/100,000 in our Asian/Pacific Islander women, 7.4/100,000 in our white women, 9.9/100,000 in our black women and 11.3/100,000 in our Hispanic women [53]; all rates lower than can be accomplished by the three dose on-time HPV4 series alone: 14/100,000 [54]. This inefficiency rate needs to be included in cost effectiveness models to determine whether “any number of doses is better than no doses”.

## References

[1] CDC (2013) Vaccine Price List. Available: http://www.cdc.gov/vaccines/programs/vfc/awardees/vaccine-management/price-list/index.html. Accessed 2013 Feb 10.

[2] Goldie SJ, O’Shea M, Diaz M, Kim SY (2008) Benefits, cost requirements and cost-effectiveness of the HPV16,18 vaccine for cervical cancer prevention in developing countries: policy implications. Reprod Health Matters. 16: 86–96. 10.1016/S0968-8080(08)32409-4 19027626

[3] United States Securities and Exchange Commission (2011) Merck &Co: Strategic National Stockpile of Gardasil®. 10-K SEC. Available: http://phx.corporate-ir.net/External.File?item=UGFyZW50SUQ9ODM1MDV8Q2hpbGRJRD0tMXxUeXBlPTM=&t=1. Accessed 2013 Feb 1.

[4] FDA news (2008) CDC Purchased 10 Million Doses of Gardasil®. Available: http://www.fdanews.com/newsletter/article?articleId=103084&issueId=11210. Accessed 2013 Feb 1.

[5] Centers for Disease Control and Prevention (2012) National and State Vaccination Coverage Among Adolescents Aged 13 Through 17 Years – United States, 2011. MMWR. 61: 671–677. 22932301

[6] Verdenius I, Harper DM, Harris GD, Griffith RS, Wall J, et al (2013) Predictors of Three Dose On-Time Compliance with HPV4 Vaccination in a Disadvantaged, Underserved, Safety Net Population in the US Midwest. PLoS ONE. 8: e71295. 10.1371/journal.pone.0071295 PMC3738587 23951123

[7] Kessels SJ, Marshall HS, Watson M, Braunack-Mayer AJ, Reuzel R, et al (2012) Factors associated with HPV vaccine uptake in teenage girls: a systematic review. Vaccine. 30: 3546–56. 10.1016/j.vaccine.2012.03.063 22480928

[8] Gold R, Naleway AL, Jenkins LL, Riedlinger KK, Kurosky SK, et al (2011) Completion and timing of the three-dose human papillomavirus vaccine series among adolescents attending school-based health centers in Oregon. Prev Med. 52: 456–8. 10.1016/j.ypmed.2011.04.010 21539853

[9] Niccolai LM, Mehta NR, Hadler JL (2011) Racial/Ethnic and poverty disparities in human papillomavirus vaccination completion. Am J Prev Med. 41: 428–33. 10.1016/j.amepre.2011.06.032 21961471

[10] Rouzier R, Giordanella JP (2010) Coverage and compliance of Human Papilloma Virus vaccines in Paris: demonstration of low compliance with non-school-based approaches. J Adolesc Health. 47: 237–41. 10.1016/j.jadohealth.2010.04.006 20708561

[11] Schluterman NH, Terplan M, Lydecker AD, Tracy JK (2011) Human papillomavirus (HPV) vaccine uptake and completion at an urban hospital. Vaccine. 29: 3767–72. 10.1016/j.vaccine.2011.03.032 21440038

[12] Cook RL, Zhang J, Mullins J, Kauf T, Brumback B, et al (2010) Factors associated with initiation and completion of human papillomavirus vaccine series among young women enrolled in Medicaid. J Adolesc Health. 47: 596–9. 10.1016/j.jadohealth.2010.09.015 21094437

[13] Chao C, Velicer C, Slezak JM, Jacobsen SJ (2009) Correlates for completion of 3-dose regimen of HPV vaccine in female members of a managed care organization. Mayo Clin Proc. 84: 864–70. 10.4065/84.10.864 PMC2755805 19797775

[14] Tan W, Viera AJ, Rowe-West B, Grimshaw A, Quinn B, et al (2011) The HPV vaccine: are dosing recommendations being followed? Vaccine. 29: 2548–54. 10.1016/j.vaccine.2011.01.066 21300098

[15] Chou B, Krill LS, Horton BB, Barat CE, Trimble CL (2011) Disparities in human papillomavirus vaccine completion among vaccine initiators. Obstet Gynecol. 118: 14–20. 10.1097/AOG.0b013e318220ebf3 PMC4696007 21691158

[16] Hirth JM, Tan A, Wilkinson GS, Berenson AB (2012) Completion of the human papillomavirus vaccine series among insured females between 2006 and 2009. Cancer. 118: 5623–5629. 10.1002/cncr.27598 PMC3410047 22544681

[17] Perkins RB, Brogly SB, Adams WG, Freund KM (2012) Correlates of human papillomavirus vaccination rates in low-income, minority adolescents: a multicenter study. J Women’s Health (Larchmt). 21: 813–820. 10.1089/jwh.2011.3364 PMC3411349 22860770

[18] Dempsey A, Cohn L, Dalton V, Ruffin M (2010) Patient and clinic factors associated with adolescent human papillomavirus vaccine utilization within a university-based health system. Vaccine. 28: 989–95. 10.1016/j.vaccine.2009.10.133 PMC2887043 19925899

[19] Dempsey A, Cohn L, Dalton V, Ruffin M (2011) Worsening disparities in HPV vaccine utilization among 19–26 year old women. Vaccine. 29: 528–34. 10.1016/j.vaccine.2010.10.051 PMC3013617 21050904

[20] Dorell CG, Yankey D, Santibanez TA, Markowitz LE (2011) Human papillomavirus vaccination series initiation and completion, 2008–2009. Pediatrics. 128: 830–9. 10.1542/peds.2011-0950 22007006

[21] Neubrand TP, Breitkopf CR, Rupp R, Breitkopf D, Rosenthal SL (2009) Factors associated with completion of the human papillomavirus vaccine series. Clin Pediatr (Phila). 48: 966–9. 10.1177/0009922809337534 19483128

[22] Smith LM, Brassard P, Kwong JC, Deeks SL, Ellis AK, et al (2011) Factors associated with initiation and completion of the quadrivalent human papillomavirus vaccine series in an Ontario cohort of grade 8 girls. BMC Public Health. 11: 645. 10.1186/1471-2458-11-645 PMC3224094 21838921

[23] Widdice LE, Bernstein DI, Leonard AC, Marsolo KA, Kahn JA (2011) Adherence to the HPV vaccine dosing intervals and factors associated with completion of 3 doses. Pediatrics. 127: 77–84. 10.1542/peds.2010-0812 PMC3010090 21149425

[24] Tiro JA, Pruitt SL, Bruce CM, Persaud D, Lau M, et al (2012) Multilevel correlates for human papillomavirus vaccination of adolescent girls attending safety net clinics. Vaccine. 30: 2368–75. 10.1016/j.vaccine.2011.11.031 22108490

[25] Rubin RF, Kuttab HM, Rihani RS, Reutzel TJ (2012) Patient Adherence to Three Dose Completion of the Quadrivalent Human Papillomavirus (HPV) Vaccine in a Private Practice. J Community Health. 37: 1145–50. 10.1007/s10900-012-9581-9 22752532

[26] Dobson SR, McNeil S, Dionne M, Dawar M, Ogilvie G, et al (2013) Immunogenicity of 2 Doses of HPV Vaccine in Younger Adolescents vs 3 Doses in Young Women: A Randomized Clinical Trial. JAMA. 309: 1793–1802. 10.1001/jama.2013.1625 23632723

[27] Smolen KK, Gelinas L, Franzen L, Dobson S, Dawar M, et al (2012) Age of recipient and number of doses differentially impact human B and T cell immune memory responses to HPV vaccination. Vaccine. 30: 3572–9. 10.1016/j.vaccine.2012.03.051 22469863

[28] Neuzil KM, Canh do G, Thiem VD, Janmohamed A, Huong VM, et al (2011) Immunogenicity and reactogenicity of alternative schedules of HPV vaccine in Vietnam: a cluster randomized noninferiority trial. JAMA. 305: 1424–31. 10.1001/jama.2011.407 21486975

[29] Elbasha EH, Dasbach EJ (2010) Impact of vaccinating boys and men against HPV in the United States. Vaccine. 28: 6858–67. 10.1016/j.vaccine.2010.08.030 20713101

[30] Nelson JC, Bittner RC, Bounds L, Zhao S, Baggs J, et al (2009) Compliance with multiple-dose vaccine schedules among older children, adolescents, and adults: results from a vaccine safety datalink study. Am J Public Health. 99: S389–97. 10.2105/AJPH.2008.151332 PMC4504385 19797753

[31] Freeman HP, Wingrove BK (2005) Excess Cervical Cancer Mortality: A Marker for Low Access to Health Care in Poor Communities. Rockville, MD: National Cancer Institute, Center to Reduce Cancer Health Disparities, May 2005. NIH Pub. No. 05–5282. Available: http://crchd.cancer.gov/attachments/excess-cervcanmort.pdf. Accessed 2013 Jan 15.

[32] Scarinci IC, Garcia FA, Kobetz E, Partridge EE, Brandt HM, et al (2010) Cervical cancer prevention: new tools and old barriers. Cancer. 116: 2531–42. 10.1002/cncr.25065 PMC2876205 20310056

[33] FDA Approval Letter (2006) Human Papillomavirus Quadrivalent (Types 6, 11, 16, 18) Vaccine, Recombinant. Available: http://www.fda.gov/BiologicsBloodVaccines/Vaccines/ApprovedProducts/ucm111283.htm. Accessed 2013 Feb 1.

[34] Markowitz LE, Dunne EF, Saraiya M, Lawson HW, Chesson H, et al (2007) Quadrivalent Human Papillomavirus Vaccine: Recommendations of the Advisory Committee on Immunization Practices (ACIP). MMWR Recomm Rep. 56: 1–24. 17380109

[35] Kreimer AR, Rodriguez AC, Hildesheim A, Herrero R, Porras C, et al (2011) Proof-of-principle evaluation of the efficacy of fewer than three doses of a bivalent HPV16/18 vaccine. J Natl Cancer Inst. 103: 1444–51. 10.1093/jnci/djr319 PMC3186781 21908768

[36] StatSoft Inc. (2010) STATISTICA (data analysis software system), version 9.1. Available: www.statsoft.com.

[37] Harper DM, Vierthaler SL, Santee JA (2010) Review of Gardasil. Journal of Vaccines and Vaccination. 1: 107. 10.4172/2157-7560.1000107 PMC3690661 23805398

[38] United States Securities and Exchange Commission (2012) Merck & Co., Inc. Quarterly SEC Report. Available: http://services.corporate-ir.net/SEC.Enhanced/SecCapsule.aspx?c=73184&fid=8316959. Page 47, August, 2012.

[39] United States Securities and Exchange Commission (2012) Merck & Co., Inc. Annual SEC Report. Available: http://www.merck.com/investors/financials/annual-reports/home.html. Page 51, February, 2012.

[40] Brisson M, Van de Velde N, Boily MC (2011) Different population-level vaccination effectiveness for HPV types 16, 18, 6 and 11. Sex Transm Infect. 87: 41–3. 10.1136/sti.2010.044412 20924049

[41] Department of Health and Human Services Centers for Disease Control and Prevention (2009) Advisory Committee on Immunization Practices (ACIP) Summary Report. June 24–26, 2009. Available: http://www.cdc.gov/vaccines/acip/meetings/minutes-archive.html. Accessed 2013 Feb 1.

[42] French KM, Barnabas RV, Lehtinen M, Kontula O, Pukkala E, et al (2007) Strategies for the introduction of human papillomavirus vaccination: modeling the optimum age- and sex-specific pattern of vaccination in Finland. Br J Cancer. 96: 514–8. 10.1038/sj.bjc.6603575 PMC2360033 17245341

[43] Rosen AB, Spaulding AB, Greenberg D, Palmer JA, Neumann PJ (2009) Patient adherence: a blind spot in cost-effectiveness analyses? Am J Manag Care. 15: 626–32. 19747027

[44] Sheinfeld Gorin SN, Glenn BA, Perkins RB (2011) The human papillomavirus (HPV) vaccine and cervical cancer: uptake and next steps. Adv Ther. 28: 615–39. 10.1007/s12325-011-0045-x 21818672

[45] Grant D, Hurley J, Williams H (2012) Department of Health and Human Services. Office of Inspector General. Vaccines for Children Program: Vulnerabilities in vaccine management. OEI-04-10-00430 June 2012. Available: https://oig.hhs.gov/oei/reports/oei-04-10-00430.asp. Accessed 2013 Feb 1.

[46] Harper DM, Vierthaler SL (2011) Next Generation Cancer Protection: The Bivalent HPV Vaccine for Females. ISRN Obstet Gynecol. 2011: 457204. 10.5402/2011/457204 PMC3216348 22111017

[47] Kuehn BM (2011) Two doses of HPV vaccine may be sufficient. JAMA. 306: 1643. 10.1001/jama.2011.1480 22009089

[48] Romanowski B, Schwarz TF, Ferguson LM, Peters K, Dionne M, et al (2011) Immunogenicity and safety of the HPV-16/18 AS04-adjuvanted vaccine administered as a 2-dose schedule compared to the licensed 3-dose schedule: Results from a randomized study. Hum Vaccin. 7: 1374–86. 10.4161/hv.7.12.18322 PMC3338934 22048171

[49] Zimmerman RK, Nowalk MP, Lin CJ, Fox DE, Ko FS, et al (2010) Randomized trial of an alternate human papillomavirus vaccine administration schedule in college-aged women. J Womens Healt. 19: 1441–7. 10.1089/jwh.2009.1753 20629576

[50] Setia S, Mainzer H, Washington ML, Coil G, Snyder R, et al (2002) Frequency and causes of vaccine wastage. Vaccine. 20: 1148–56. 10.1016/s0264-410x(01)00433-9 11803076

[51] Moyer VA (2012) U.S. Preventive Services Task Force. Screening for cervical cancer: U.S. Preventive Services Task Force recommendation statement. Ann Intern Med. 156: 880–91. 10.7326/0003-4819-156-12-201206190-00424 22711081

[52] Goldie SJ, Diaz M, Kim SY, Levin CE, van Minh H, et al (2008) Mathematical Models of Cervical Cancer Prevention in the Asia Pacific Region. ICO HPV Monograph Series: Asia Pacific Regional Report. Vaccine 26S: M17–M29. 10.1016/j.vaccine.2008.06.018 18945411

[53] Centers for Disease Control and Prevention (CDC) (2012) Human papillomavirus-associated cancers - United States, 2004–2008. MMWR Morb Mortal Wkly Rep. 61: 258–61. 22513527

[54] Harper DM, Williams KB (2010) Prophylactic HPV vaccines: current knowledge of impact on gynecologic premalignancies. Discov Med. 10: 7–17. 20670593

